# Diverse Mechanisms Regulate the Surface Expression of Immunotherapeutic Target CTLA-4

**DOI:** 10.3389/fimmu.2014.00619

**Published:** 2014-12-04

**Authors:** Helga Schneider, Christopher E. Rudd

**Affiliations:** ^1^Cell Signalling Section, Division of Immunology, Department of Pathology, University of Cambridge, Cambridge, UK

**Keywords:** CTLA-4, trafficking, TRIM, LAX, Rab8

## Abstract

T-cell co-receptor cytotoxic T-cell antigen-4 (CTLA-4) is a critical inhibitory regulator of T-cell immunity and antibody blockade of the co-receptor has been shown to be effective in tumor immunotherapy. Paradoxically, the majority of CTLA-4 is located in intracellular compartments from where it is transported to the cell surface and rapidly internalized. The intracellular trafficking pathways that control transport of the co-receptor to the cell surface ensures the appropriate balance of negative and positive signaling for a productive immune response with minimal autoimmune disorders. It will also influence the degree of inhibition and the potency of antibody checkpoint blockade in cancer immunotherapy. Current evidence indicates that the mechanisms of CTLA-4 transport to the cell surface and its residency are multifactorial involving a combination of immune cell-specific adapters such as TRIM and LAX, the small GTPase Rab8 as well as generic components such as ARF-1, phospholipase D, and the heterotetrameric AP1/2 complex. This review covers the recent developments in our understanding of the processes that control the expression of this important co-inhibitory receptor for the modulation of T-cell immunity. Interference with the processes that regulate CTLA-4 surface expression could provide an alternate therapeutic approach in the treatment of cancer and autoimmunity.

## Introduction

The co-receptor cytotoxic T lymphocyte antigen-4 (CTLA-4; CD152) is a central inhibitory regulator of T-cell proliferation and expansion (1–5). Its dampening effect on the activation process limits and terminates T-cell responses, and as such is important for regulating peripheral T-cell tolerance and autoimmunity. A negative role for the co-receptor in the control of proliferation and autoimmunity was initially observed in the striking phenotype of the *Ctla4−/−* mouse (6, 7). These mice show polyclonal T-cell activation or autoproliferation that leads to massive tissue infiltration and early lethality. An additional linkage of single-nucleotide polymorphisms (SNPs) in the region of CTLA-4 were subsequently found associated with a variety of autoimmune disorders that include type 1 diabetes, coeliac disease, myasthenia gravis, Hashimoto’s thyroiditis, systemic lupus erythematosus (SLE), and Wegener’s granulomatosis (8–12). Immune dysregulation in human subjects has also been reported recently with heterozygous germline mutations in CTLA-4 (13). This plurality of associated autoimmune disorders in human beings has pointed to a central role for the co-inhibitory receptor as a general regulator of the threshold signals needed for T-cell activation. Under normal conditions, the inhibition of signaling events protects against responses to lower affinity self-antigen while allowing responses to higher affinity foreign antigen. In this sense, minor changes in the surface expression of the co-receptor are thought to have significant effects on responses to autoantigen. Ipilimumab, a humanized anti-CTLA-4 checkpoint blockade antibody, has also been found impressively effective in the treatment of various tumors such as melanoma and small cell lung carcinomas (14, 15). Combined therapy with antibodies against another negative co-receptor PD-1 (programmed cell death-1) has been found to co-operate with anti-CTLA-4 to induce even more striking response rates (16).

Given that minor changes in the surface expression of the co-receptor are expected to have significant effects on responses to autoantigen and in cancer immunotherapy, it is important to understand the mechanisms that determine the expression of CTLA-4 on T-cells. This includes the intracellular pathways that determine the transport or trafficking of CTLA-4 to the cell surface as well as events that regulate its residency on the surface and endocytosis. Paradoxically, CTLA-4 is primarily located in intracellular compartments from where it is rapidly recycled to the cell surface. Only small amounts of the co-receptor can be detected on the cell surface at any given time, even when optimally expressed following T-cell activation. This review covers the recent developments in our understanding of the events that control the transport and expression of CTLA-4 to the cell surface for the modulation of T-cell immunity.

## Structure and Function of CTLA-4

CTLA-4 was one of the first and most extensively investigated co-inhibitory receptor of the immune system (17). The CTLA-4 gene consists of four exons: exon 1 contains the leader peptide sequence, exon 2 the ligand binding site, exon 3 encodes the transmembrane region, and exon 4 the cytoplasmic tail (18). Differential splicing of the CTLA-4 transcript results in a full-length transmembrane form (exons 1–4), soluble CTLA-4 (lacking exon 3), and a transcript encoding only for exons 1 and 4 (19, 20). Murine T-cells also express a ligand-independent CTLA-4 (liCTLA-4) containing exons 1, 3, and 4 (12). Although liCTLA-4 lacks the MYPPPY ligand binding domain, it strongly inhibits T-cell responses and, compared to full-length CTLA-4, its expression is elevated in regulatory and memory T-cells from diabetes resistant NOD mice (21).

CTLA-4 is structurally related to CD28 with some 30% sequence homology (22). It was first described as the product of the *Ctla4* gene located at chromosome 1 (mouse) or 2 (human being) and is preferentially expressed in activated cytolytic T-cells (17). Subsequently, it was found to be expressed in all activated T-cells and used as an early activation marker. mRNA for CTLA-4 can be detected as early as 1 h post-activation with maximum expression between 24 and 36 h, the time when CTLA-4 is detectable on the cell surface (23, 24). In contrast to full-length CTLA-4, ligand-independent CTLA-4 is expressed in resting cells, but downregulated during early activation (21). Like CD28, CTLA-4 binds to ligands CD80 and CD86 but with greater avidity (25, 26). The same signature MYPPPY motif for binding is found in both co-receptors (27). The higher avidity of CTLA-4 for CD80 is due to the binding of one CTLA-4 homodimer to two CD80 molecules (28, 29) resulting in the formation of a stable CTLA-4/CD80 lattice structure in the immunological synapse (IS). This interaction may disturb the assembly of key signaling proteins needed for CD28 co-stimulation.

As mentioned, the importance of CTLA-4 in maintaining peripheral tolerance and homeostasis was first demonstrated with the autoimmune phenotype of CTLA-4-deficient mice. These mice show polyclonal T-cell activation leading to massive tissue infiltration and early lethality (6, 7). Further, SNPs of the human CTLA-4 gene have been implicated in the susceptibility to autoimmune disorders such as type I diabetes, rheumatoid arthritis, and multiple sclerosis (12). However, it is still unknown how and whether SNPs affect CTLA-4 function (i.e., intracellular trafficking, surface expression, dimerization). The soluble form of CTLA-4 has been linked to autoimmune diseases. High concentrations of soluble CTLA-4 can be detected in patients with various autoimmune diseases (30–32).

Unlike in the case of conventional T-cells (Tconv), suppressive regulatory T-cells (Tregs) express CTLA-4 constitutively on the cell surface. In fact, the pool of intracellular CTLA-4 seen in activated Tconv is less apparent in Tregs, a finding that may account for its constitutively high level of surface expression (33). Given this fact, it is not surprising that CTLA-4 is intimately linked to the regulation of Treg suppressor function (34, 35). Mechanisms that have been reported to account for Treg function include the secretion of the suppressive cytokines IL-10, IL-35, and TGF-β (36), secretion of cytolytic granules containing granzyme and perforin as well as competition with conventional responder T-cells for CD80 and CD86 on antigen-presenting cells (APCs) (37, 38). Given its higher avidity for binding to CD80/86, CTLA-4 would block the availability of CD80 and CD86 for an interaction with Tconv. While CTLA-4 on Tconv induces their motility and limits their contact time with APCs, resulting in hypoactivation of these cells, CTLA-4 on Tregs does not influence their dwell times and, therefore, would allow the co-receptor to interfere with CD80/86 presentation to CD28 (39).

## CTLA-4 and Tumor Immunotherapy

An exciting development over the past few years has been the use of anti-CTLA-4 in so-called checkpoint blockade in the treatment of cancers. These human studies originated from earlier mouse tumor models, which demonstrate that blockade of CTLA-4-mediated inhibition leads to enhancement of T-cell responses in tumor immunotherapy (40). Early human studies with limited numbers of patients (41–44) were expanded to larger phase III studies showing response rates as high as 30% on melanoma, small cell lung, and renal carcinoma (14–16). These studies led to the generation of antibodies to human CTLA-4, ipilimumab, and tremelimumab (45). Ipilimumab has been approved as monotherapy for the treatment of advanced melanoma. They have shown synergistic anti-tumor activity when utilized with vaccines, chemotherapy, and radiation (14). CTLA-4 antibodies have also induced a reversible occurrence of immune-related adverse events (IRAE) such as colitis, dermatitis, or endocrinopathies (46). The exact mechanism by which anti-CTLA-4 mediates enhanced anti-tumor reactivity is not clear, but may involve a combination of effects involving the lowering of the threshold needed to activate T-cells, a reduction in the number of Tregs, the reduced release of the suppressive factor indoleamine 2,3-dioxygenase (IDO) as well as broadening the peripheral T-cell receptor repertoire (47, 48). In certain instances, co-operation with interleukin-2 treatment has also been observed (49). More recently, antibodies against PD-1, another inhibitory co-receptor, have also demonstrated remarkable clinical anti-tumor activity against melanoma and other solid tumors (50). Further, the combination of anti-CTLA-4 and PD-1 antibodies achieved an even more effective anti-tumor response (16, 51). CTLA-4 engagement with CD80/CD86 attenuates the early activation of naïve and memory T-cell, whereas PD-1 is mainly thought to modulate T-cell effector functions in peripheral tissues via binding to PD-L1 and PD-L2 (52). Since CTLA-4 and PD-1 regulate immune responses in a non-redundant fashion, combined blockade of both pathways may achieve more effective anti-tumor activity.

## Mechanisms of CTLA-4-Mediated Inhibition

Despite the importance of CTLA-4 to autoimmunity and anti-tumor immunotherapy, the actual mechanisms responsible for its function are unknown. Much debate has focused on whether CTLA-4 inhibits T-cell responses by cell-extrinsic or -intrinsic mechanisms. Cell intrinsic mechanisms would reflect direct effects of the co-receptor on the expressing cell (i.e., signal transduction), while cell-extrinsic effects relate to the regulation of function via a distal cell or cytokine. Both mechanisms have been implicated in the *in vivo* function of CTLA-4 (53). A cell-extrinsic pathway for CTLA-4 was first described by Bachman and coworkers who found that Rag2-deficient mice reconstituted with a mixture of wild-type and CTLA-4-deficient bone marrow cells failed to develop autoimmune disease, while the transfer of *Ctla4−/−* bone marrow cells alone transferred disease (54). Cell-intrinsic and non-cell-autonomous (i.e., cell extrinsic) actions of CTLA-4 have been reported to operate to maintain T-cell tolerance to self-antigen (53). In agreement with this observation, Thompson and coworkers found that the loss of the cytoplasmic tail of CTLA-4 (i.e., cell intrinsic) affected the onset of disease as well as differences in T-cell infiltration. These findings suggested possible differences for cell intrinsic versus extrinsic mechanisms in the autoproliferative versus migratory aspects of CTLA-4 inhibition (55). Others have emphasized the importance of cell-extrinsic mechanisms on both Tconv and Tregs, although this may vary with antigen dose and the model examined (56). It is possible that CTLA-4 utilizes different pathways for inhibition in different contexts or niches of the immune system.

**Figure 1:**
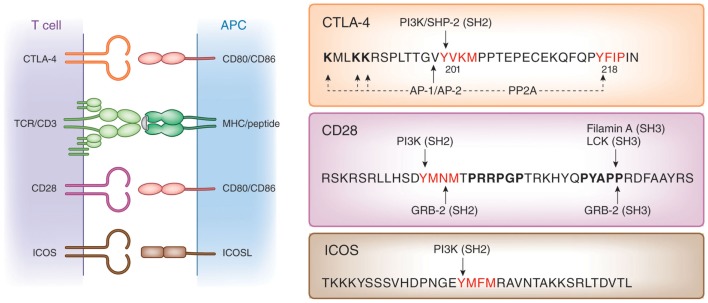
**Structure of co-receptors. Left panel**: CTLA-4 and CD28 bind to the same natural ligands CD80/CD86 via the MYPPPY motif, whereas ICOS binds to ICOSL via the FDPPPF motif. **Right panel**: structure of the cytoplasmic domains of human CTLA-4, CD28, and ICOS. The cytoplasmic domains of these co-receptors have a common YxxM motif, which binds to the SH2 domain of the p85 subunit of phosphatidylinositol 3-kinase (PI3K). CTLA-4 has a unique YVKM motif, which binds to the SH2 domain of the tyrosine phosphatase SHP-2. In its non-phosphorylated form, it associates with the clathrin adapters AP-1 and AP-2. The serine/threonine phosphatase PP2A binds to the lysine rich motif and the tyrosine 218 (Y_218_FIP). The asparagine in the YMNM motif of CD28 is needed for Grb-2 SH2 domain binding, whereas the distal proline motif allows for binding of the SH3 domains of Grb-2, the protein tyrosine kinase p56lck, and Filamin A.

Cell intrinsic pathways include modulation of TCR signaling by phosphatases SHP-2 and PP2A (57), inhibition of ZAP-70 microcluster formation (58), and altered IS formation (59), as well as interference with the expression or composition of lipid rafts on the surface of T-cells (60–63). Like CD28 and ICOS, CTLA-4 possesses a small cytoplasmic tail containing, apart from its C-terminal YFIP motif, a YxxM consensus motif common of all three co-receptors (64) (Figure 1). Several intracellular proteins including the lipid kinase phosphatidylinositol 3-kinase (PI3K) (65), the phosphatase SHP-2 (4, 57, 66, 67) and clathrin adapter proteins AP1 and AP2 (68–70) have been reported to bind to the YVKM motif. The phosphatase PP2A has also been reported to interact with the cytoplasmic tail of CTLA-4 via the lysine rich motif and via the tyrosine residue at position 218 (71). CTLA-4-mediated phoshorylation of Akt is abrogated by the PP2A inhibitor okadaic acid (72). By contrast, PD-1 signaling inhibits Akt phosphorylation by preventing CD28-mediated activation of PI3K that is dependent on the immunoreceptor tyrosine-based switch motif (ITSM) located in its cytoplasmic tail (72).

Cell-extrinsic mechanisms include CTLA-4 engagement of CD80/CD86 on dendritic cells (DCs) that can induce the release of IDO (73, 74). This enzyme catalyzes the degradation of the amino acid l-tryptophan to *N*-formylkynurenine leading to the depletion of tryptophan, which in turn can halt the growth of T-cells. Although IDO has been implicated in certain immune responses (75, 76), it is unlikely to solely account for the phenotype of the *Ctla4−/−* mouse since IDO-deficient mice fail to develop autoimmunity (77). CTLA-4 has also been reported to increase the production of the immunosuppressive cytokine TGF-β (78); however, TGF-β-deficient mice differ from CTLA-4-deficient mice in the severity of the autoimmune phenotype (79). The multiorgan inflammatory syndrome can be inhibited by depletion of the activated CD4 positive T-cells leading to prolonged survival; however, the TGF-β-deficient mice eventually die of myeloid hyperplasia (80).

Not unexpectedly, Tregs play a major role in cell-extrinsic regulation. Both CTLA-4-deficient and FoxP3-deficient mice exhibit a short life span due to massive lymphoproliferation (LP) and a systemic autoimmune-like syndrome (6, 7, 81). The conditional loss of CTLA-4 on FoxP3 expressing cells delayed the onset of disease to 7–10 weeks, rather than to 3–4 weeks observed in *Ctla4−/−* mice (82, 83). This indicated that Tregs help control the development of the *Ctla4−/−* phenotype and that both CTLA-4 and FoxP3 on the same cell subset are essential to fully prevent LP disease. However, while Tregs help to control the onset of disease, the fact that the mice still die suggests that another factor is causally responsible for the onset of the autoimmune-like syndrome.

The mechanism by which CTLA-4 facilitates Treg function is unclear but may involve the occupancy of CD80 and CD86 on DCs (82, 83). Trans-endocytosis or the removal of CD80 or CD86 from the surface of DCs may also occur (83, 84). Since both Tregs and Tconv can mediate this removal, it is uncertain whether this property can be the primary mechanism to account for Treg suppression. On the other hand, in certain models, some groups have claimed that the mere expression of CTLA-4 on either subset is sufficient to mediate cell-extrinsic suppression (33, 56). Tregs with higher CTLA-4 levels are able to be more effective in blocking or trans-endocytosis than Tconv cells. In this context, recent elegant work has shown that CTLA-4 can bind to the protein kinase C isoform η (PKC-η) in Tregs (and not Tconv cells) and that defective activation of CTLA-4-PKC-η with another complex in PKC-η-deficient cells correlates with the reduced depletion of CD86 from APCs (85). CTLA-4-associated SHP-1/2 and PP2A are not recruited to the IS of Tregs (85, 86).

Another model involves a combination of cell-intrinsic and -extrinsic effects related to altered T-cell adhesion and motility (87, 88). We and others have shown that CTLA-4 ligation activates the small GTPase Rap-1 (89, 90). Rap1 is a key molecule involved in the activation of integrins such as lymphocyte function-associated antigen-1 (LFA-1). In this model, CTLA-4 is a motility activator and augments T-cells adhesion (88, 90). Significantly, anti-CTLA-4 alone was able to induce motility of primary T-cells and cell lines (58, 88). As a motility activator, CTLA-4 bypasses the TCR-mediated stop-signal that is needed for stable interactions between T-cells and APCs. This provided an alternate mechanism to account for the dampening effect of CTLA-4 on T-cell activation and has been confirmed in several different models (87, 88, 90–95). In this model, the cell intrinsic pathway involves activation of Rap1 and the ligation efficiency of the TCR on Tconvs, while the cell-extrinsic pathway involves the regulation of T-cell binding to APCs. The reversal of the stop-signal by CTLA-4 was exclusively seen on Tconv and not Tregs (39).

## CTLA-4 Trafficking from the *Trans*-Golgi Network to the Cell Surface

**Figure 2:**
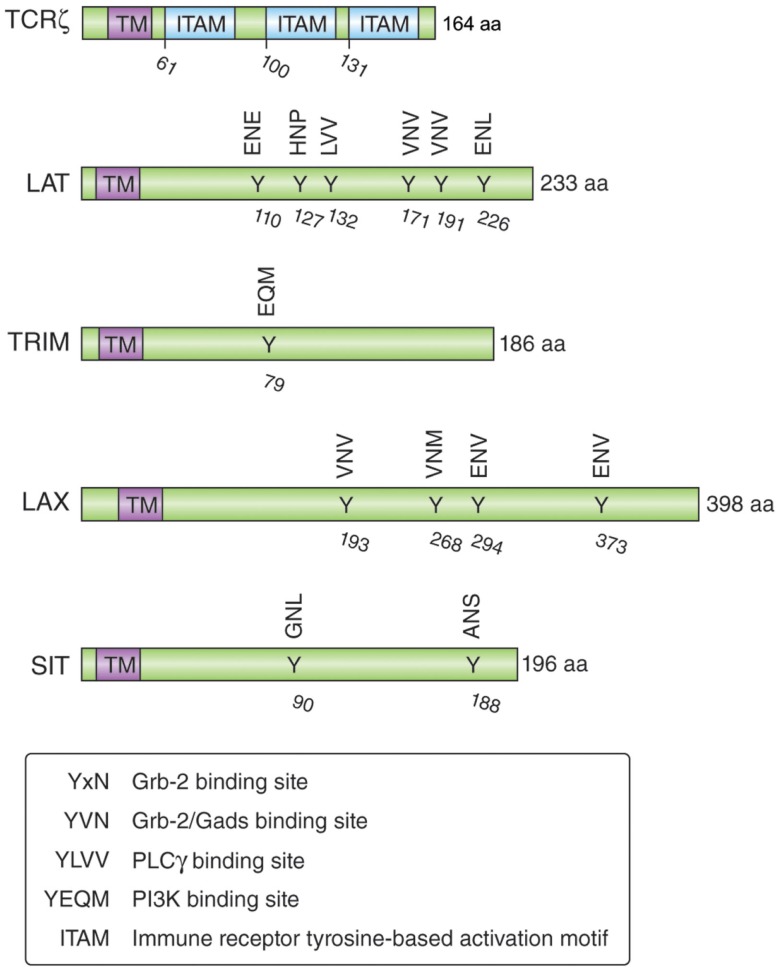
**Schematic structure of the transmembrane adapters TCR***ζ***, LAT, TRIM, LAX, and SIT with their binding motifs for Grb-2, Gads, PI3K, and PLC***γ*****.

Understanding the mechanisms by which CTLA-4 is transported to the cell surface will be the key to the development of novel strategies to increase or decrease its expression and functional effects. An ability to interfere with the trafficking pathways in T-cells would provide an alternate approach to the use of biologics such as anti-CTLA-4 antibodies. Previous studies have demonstrated the need of calcium for the release of CTLA-4 from the *Trans-*Golgi network (TGN) to the cell surface (69, 96), while other studies have implicated more generic processes involving the GTPase ADP ribolysation factor-1 (ARF-1) and phospholipase D (PLD) (97). However, these pathways are also involved in the transport of other non-lymphoid receptors and thus are not specific for CTLA-4. In this context, it has been demonstrated that TCRzeta (TCRζ) plays a central role in transporting the TCR to the cell surface (98, 99). TCRζ is a member of the type III transmembrane adapter proteins (TRAPs), which possess a short extracellular domain, a single transmembrane domain, and a relatively long cytoplasmic tail with several tyrosine phosphorylation sites (100, 101) (Figure 2). Based on the TCRzeta model, we hypothesized that this family of transmembrane proteins might play a general role in the transport of surface receptors. Other members of the TRAP family include TRIM (T-cell receptor-interacting molecule), LAX (linker for activation of X cells), SIT (SHP2 interacting TRAP), and LAT (linker for activation of T-cells) (100, 101). As in the case of the TCRζ, they are preferentially expressed in immune cells, but most of them lack the signaling effects seen with the TCRzeta chain. For example, they lack the immunoreceptor tyrosine-based activation motifs (ITAMs) needed for binding to the protein tyrosine kinase ZAP-70. Instead, they are enriched in binding sites for PI-3K and Grb-2/Gads (102, 103).

TRIM is highly expressed in thymocytes and CD4 positive T-cells and forms a disulfide-linked homodimer (104). It possesses three tyrosine-based motifs in its cytoplasmic tail (two YxxL motifs and one YxxM motif), where the YxxM motif binds to the p85 subunit of PI3 kinase (102) (Figure 2). Initial TRIM overexpression studies in Jurkat T-cells suggested that TRIM upregulates the surface expression of the TCR and mediates increased calcium release after TCR ligation (105). However, T-cell development, TCR surface expression, and signaling events induced by TCR ligation are not impaired in TRIM-deficient mice (104). LAX is expressed as a monomer and possesses a longer cytoplasmic tail (398 aa versus 186 aa), which contains eight tyrosine-based motifs; five of them represent binding sites for Grb-2/Gads (103). LAX negatively impairs TCR signaling events as shown with LAX overexpression studies in Jurkat T-cells leading to inhibition of p38 and NFAT/AP-1 (106). Although LAX deficiency does not impair lymphocyte development, T- and B-cells are hyperresponsive upon TCR or BCR ligation and show increased cell survival (107). Mutation studies of the tyrosine-based motifs revealed the importance of the binding signaling proteins (Grb-2, Gads, and PI3K) in the inhibitory function of LAX (103).

**Figure 3:**
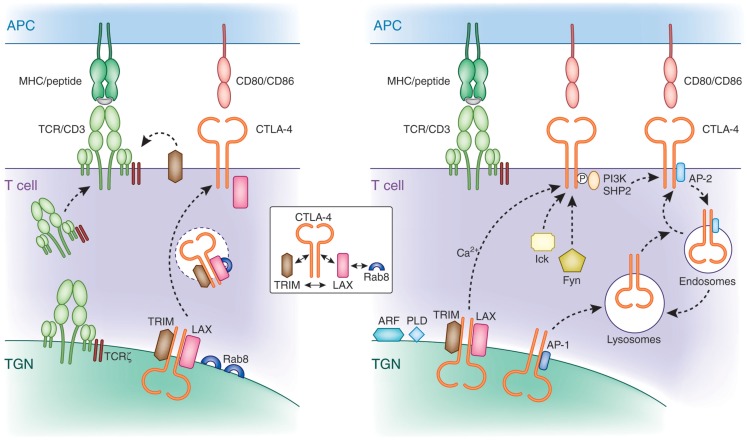
**Mechanisms of CTLA-4 trafficking. Left panel**: CTLA-4 forms a multimeric complex composed of TRIM, LAX, and Rab8 for post-Golgi transport to the cell surface. TRIM and LAX bind to the cytoplasmic tail of CTLA-4, while LAX binds via its N-terminus to active GTP-Rab8 (inset). TRIM requires LAX for binding to Rab8 in a complex. LAX functions as a central coordinator by bridging Rab8 with the other LAX-associated proteins TRIM and CTLA-4. This multimeric complex facilitates the transport of newly translated CTLA-4 to the cell surface. The transmembrane adapters TRIM and LAX play a role for CTLA-4 transport similar to the TCRzeta chain, which resides in the TGN and surrounding vesicles and is needed for the association with the hexameric TCR/CD3 in the TGN for proper and efficient cell surface expression. **Right panel**: non-phosphorylated CTLA-4 associates with the clathrin adapter complex AP-1 and AP-2. AP-1 regulates trafficking of CTLA-4 by shuttling the receptor from the TGN to lysosomes. In activated cells, newly synthesized CTLA-4 is transported to the cell surface, which is facilitated by the TRIM/LAX/Rab8 complex (left panel) and by more generic factors such as ARF-1, PLD, and calcium. Phosphorylation of CTLA-4 by Fyn and Lck recruits PI3K and SHP-2. Dephosphorylated CTLA-4 binds to AP-2 leading to the internalization of CTLA-4 to intracellular compartments such as endosomes and lysosomes from where it can recycle to the cell surface.

Initial shRNA knockdown and overexpression studies demonstrated that TRIM facilitates the transport of CTLA-4 to the cell surface (108, 109). TRIM specifically co-precipitated CTLA-4, but not other T-cell co-receptors such as CD28. Overexpression of TRIM potentiated CTLA-4 expression due to increased release to the cell surface, which in turn led to increased suppression of T-cell activation. Subsequently, LAX was also found to bind, co-localize, and facilitate CTLA-4 transport to the cell surface (110). CTLA-4 binding to TRIM and LAX was specific in that it did not associate with LAT. These data indicate that TRIM and LAX, both immune-specific type III proteins, bind to CTLA-4 to facilitate its transport to the cell surface (Figure 3).

Strikingly, downregulation of TRIM and LAX markedly reduced the presence of CTLA-4 expressing TGN proximal vesicles. This observation suggested a connection between CTLA-4 binding to TRIM/LAX and the budding of CTLA-4 positive vesicles from the TGN needed for transport to the cell surface. Further, these findings resemble that found for the transport of the TCR/CD3 complex to the cell surface (111). TCRζ, which resides in the TGN and surrounding vesicles needs to associate with the hexameric TCR/CD3 in the TGN for proper and efficient cell surface expression. Rab proteins are members of the Ras superfamily and regulate protein transport of the secretory and endocytic pathway (112, 113). They are active in a GTP-bound state and become inactive with the conversion of GTP to GDP. Among the different members of the Rab family, Rab8 has been shown to mediate the trafficking of newly synthesized proteins from the TGN to the plasma membrane (114), whereas other family members mediate transport of proteins among other intracellular organelles such as endoplasmatic reticulum, endosomes, and lysosomes. In addition, Rab8 alters the reorganization of actin and microtubules, as well as directing membrane transport to cell surfaces (115, 116). Activation of Rab8 can lead to cell protrusions, whereas its depletion promotes the formation of actin stress fibers (117, 118). The α_2_β and β_2_-adrenergic receptors have been described to bind Rab8 for transport to the plasma membrane (119). However, until recently, despite its high expression in T-cells, no immune cell-specific binding effectors of Rab8 have been identified.

A recent study by Banton et al. showed that the transmembrane adaptor LAX bound to the active form of Rab8, while at the same time also associated with CTLA-4 and TRIM (110). By contrast, CTLA-4 and TRIM failed to bind to Rab8. These findings demonstrate that CTLA-4 interacts with a protein complex in which TRIM and LAX bind to the co-receptor, TRIM and LAX to each other, and LAX to Rab8 (Figure 3, see inset). Importantly, disruption of LAX-Rab8 binding profoundly reduced the formation of CTLA-4 containing vesicles proximal of the TGN as well as the expression of CTLA-4 on the cell surface. The reduction in CTLA-4 expression resulted in augmented immune responses. Overall, given the established role of Rab8 as a molecule that mediates intracellular trafficking of proteins from the TGN to the plasma membrane, its association with CTLA-4 provides a pathway for the control of CTLA-4 surface expression.

Altogether, the TRIM-LAX-Rab8 connection to CTLA-4 trafficking to the cell surface will operate in co-operation with other generic processes. The ADP ribosylation factor (ARF) family GTPases and PLD are needed for the budding of vesicles at the Golgi apparatus (120–122). As in the case of Rab8, ARFs are members of GTP binding proteins of the Ras superfamily. There are six conserved ARF proteins in mammalian cells and are well established regulators of vesicle trafficking and actin re-modeling. In particular, ARF1 is involved in the regulation of vesicle transport in the TGN and the activation of PLD. PLD hydrolyzes phosphatidylcholine generating phosphatidic acid (PA) and choline. Further, the hydrolyzation of PA generates diacylglycerol, which, in addition to its signaling role, has a functional role in membrane modulation (123, 124). Dominant negative mutants of ARF-1 or PLD inhibit the release of CTLA-4 to the cell surface (97). However, unlike Rab8, none have been reported to associate with CTLA-4. Most likely, other key regulators of trafficking (i.e., SNAREs) are also needed for CTLA-4 expression. These mechanisms have been described for many surface expressed receptors and are not specific for CTLA-4.

## Recycling of CTLA-4

Following cell surface expression, CTLA-4 is rapidly internalized and again recycled to the plasma membrane of T-cells. This presumably occurs due to control of the inhibitory effects of CTLA-4 on the immune response (i.e., needs to be tightly regulated). The one exemption is Tregs, which show constitutively surface expressed CTLA-4 (24, 125). Rapid removal of CTLA-4 from the cell surface is mediated by the heterotetrameric adapter protein AP2 via clathrin-dependent internalization (68–70). AP-2 binding is regulated by the phosphorylation of the YVKM motif in the cytoplasmic tail of CTLA-4 (126, 127). Phosphorylation of CTLA-4 by protein tyrosine kinases p56lck, p59fyn, and Rlk (128, 129) promotes binding to PI3K or SHP-2 leading to the production of phosphatidylinositol (3,4,5)-triphosphate (D3 lipids) or dephosphorylation of tyrosine residues on substrates such as ITAMs in the TCR/CD3 complex and ZAP-70 (130). AP-2 binding to CTLA-4 is inhibited by the phosphorylation of the YVKM motif. Instead, once the recruitment and engagement of PI 3K is complete, CTLA-4 is dephosphorylated exposing the non-phosphorylated TGVYVKM motif. Binding of AP-2 generally involves the independently folded appendage domains of the large α (α_1_ or α_2_ isoform) and β_2_ subunits, each separated from the heterotetrameric adapter core by a flexible hinge (131). Its binding to CTLA-4 mediates the internalization of the co-receptor from the cell surface to endosomal and lysosomal compartments. Golgi-associated CTLA-4 also binds to the heterotetrameric AP-1 via the same motif (69) where it shuttles the receptor from the TGN to lysosomes. Further, upon TCR stimulation, CTLA-4 containing secretory lysosomal vesicles are released to the cell membrane resulting in increased CTLA-4 surface expression (132). Further, under conditions of T-cell inactivation, CTLA-4 colocalizes with the TCR to lipid rafts in the IS (61). The polarized release of CTLA-4 toward the site of TCR engagement has been correlated with a repositioning of the microtubule organizing center (MTOC) in T-cells (96, 133). The extent of CTLA-4 surface expression is determined by the strength of the TCR signal (133). In contrast to full-length CTLA-4, ligand-independent CTLA-4 (lacking exon 2 encoding the ectodomain including the MYPPPY motif needed for CD80/86 binding) expressed in resting mouse T-cells is downregulated during activation (21). Also, compared to activated effector T-cells, CTLA-4 is considerably longer retained on the surface of memory T-cells (134). The molecular basis for the different expression levels of CTLA-4 in both cell types remains to be established. Intracellular trafficking to the cell surface as well as endocytosis and recycling determine the overall level of CTLA-4 on the surface of T-cells.

## Summary

Optimal regulation of CTLA-4 surface expression is crucial for the balance of stimulatory and inhibitory signals to elicit proper immune responses. Minor changes in surface expression levels could have major effects on the outcome of T-cell activation. Levels of surface expressed CTLA-4 are regulated by endocytosis, recycling, and newly synthesized CTLA-4. Besides generic factors (i.e., ARF-1, PLD, SNAREs) needed for transport of multiple receptors to the cell surface, the recently identified CTLA-4-TRIM-LAX-Rab8 complex is specific in facilitating CTLA-4 transport to the cell surface. This finding is of potential importance for the development of new therapeutics that will be designed to enhance anti-tumor immunity or to increase expression in the control of autoimmune disease. Cell permeable peptides (CPP) and/or siRNA targets of immune cell trafficking adapters TRIM or LAX could provide an alternate therapy especially for patients with severe IRAE during treatment with CTLA-4 antibodies. Further, a combination of anti-PD-1 antibodies and CPP could achieve an even more effective anti-tumor response.

## Conflict of Interest Statement

The authors declare that the research was conducted in the absence of any commercial or financial relationships that could be construed as a potential conflict of interest.

## References

[1] Brunner MC Chambers CA Chan FK Hanke J Winoto A Allison JP. CTLA-4-mediated inhibition of early events of T cell proliferation. J Immunol (1999) 162:5813–20. 10229815

[2] Chambers CA Kuhns MS Egen JG Allison JP. CTLA-4-mediated inhibition in regulation of T cell responses: mechanisms and manipulation in tumor immunotherapy. Annu Rev Immunol (2001) 19:565–94. 10.1146/annurev.immunol.19.1.565 11244047

[3] Krummel MF Allison JP. CTLA-4 engagement inhibits IL-2 accumulation and cell cycle progression upon activation of resting T cells. J Exp Med (1996) 183:2533–40. 10.1084/jem.183.6.2533 8676074 PMC2192613

[4] Lee KM Chuang E Griffin M Khattri R Hong DK Zhang W Molecular basis of T cell inactivation by CTLA-4. Science (1998) 282:2263–6. 10.1126/science.282.5397.2263 9856951

[5] Walunas TL Lenschow DJ Bakker CY Linsley PS Freeman GJ Green JM CTLA-4 can function as a negative regulator of T cell activation. Immunity (1994) 1:405–13. 10.1016/1074-7613(94)90071-X 7882171

[6] Tivol EA Borriello F Schweitzer AN Lynch WP Bluestone JA Sharpe AH. Loss of CTLA-4 leads to massive lymphoproliferation and fatal multiorgan tissue destruction, revealing a critical negative regulatory role of CTLA-4. Immunity (1995) 3:541–7. 10.1016/1074-7613(95)90125-6 7584144

[7] Waterhouse P Penninger JM Timms E Wakeham A Shahinian A Lee KP Lymphoproliferative disorders with early lethality in mice deficient in Ctla-4. Science (1995) 270:985–8. 10.1126/science.270.5238.985 7481803

[8] Kristiansen OP Larsen ZM Pociot F. CTLA-4 in autoimmune diseases – a general susceptibility gene to autoimmunity? Genes Immun (2000) 1:170–84. 10.1038/sj.gene.6363655 11196709

[9] Liu J Zhang H. −1722T/C polymorphism (rs733618) of CTLA-4 significantly associated with systemic lupus erythematosus (SLE): a comprehensive meta-analysis. Hum Immunol (2013) 74:341–7. 10.1016/j.humimm.2012.12.009 23261408

[10] Pastuszak-Lewandoska D Sewerynek E Domanska D Gladys A Skrzypczak R Brzezianska E. CTLA-4 gene polymorphisms and their influence on predisposition to autoimmune thyroid diseases (Graves’ disease and Hashimoto’s thyroiditis). Arch Med Sci (2012) 8:415–21. 10.5114/aoms.2012.28593 22851994 PMC3400896

[11] Song GG Kim JH Kim YH Lee YH. Association between CTLA-4 polymorphisms and susceptibility to Celiac disease: a meta-analysis. Hum Immunol (2013) 74:1214–8. 10.1016/j.humimm.2013.05.014 23770251

[12] Ueda H Howson JM Esposito L Heward J Snook H Chamberlain G Association of the T-cell regulatory gene CTLA4 with susceptibility to autoimmune disease. Nature (2003) 423:506–11. 10.1038/nature01621 12724780

[13] Kuehn HS Ouyang W Lo B Deenick EK Niemela JE Avery DT Immune dysregulation in human subjects with heterozygous germline mutations in CTLA4. Science (2014) 345:1623–7. 10.1126/science.1255904 25213377 PMC4371526

[14] Callahan MK Postow MA Wolchok JD. Immunomodulatory therapy for melanoma: ipilimumab and beyond. Clin Dermatol (2013) 31:191–9. 10.1016/j.clindermatol.2012.08.006 23438382 PMC3653249

[15] Hodi FS O’Day SJ McDermott DF Weber RW Sosman JA Haanen JB Improved survival with ipilimumab in patients with metastatic melanoma. N Engl J Med (2010) 363:711–23 10.1056/NEJMoa1003466 20525992 PMC3549297

[16] Ott PA Hodi FS Robert C. CTLA-4 and PD-1/PD-L1 blockade: new immunotherapeutic modalities with durable clinical benefit in melanoma patients. Clin Cancer Res (2013) 19:5300–9. 10.1158/1078-0432.CCR-13-0143 24089443

[17] Brunet JF Denizot F Luciani M-F Roux-Dosseto M Suzan M-F Mattei M-G A new member of the immunoglobulin superfamily-CTLA-4. Nature (1987) 328:267–70 10.1038/328267a0 3496540

[18] Ling V Wu PW Finnerty HF Sharpe AH Gray GS Collins M Complete sequence determination of the mouse and human CTLA4 gene loci: cross-species DNA sequence similarity beyond exon borders. Genomics (1999) 60:341–55 10.1006/geno.1999.5930 10493833

[19] Huurman VA Unger WW Koeleman BP Oaks MK Chandraker AK Terpstra OT Differential inhibition of autoreactive memory- and alloreactive naive T cell responses by soluble cytotoxic T lymphocyte antigen 4 (sCTLA4), CTLA4Ig and LEA29Y. Clin Exp Immunol (2007) 150:487–93. 10.1111/j.1365-2249.2007.03513.x 17924973 PMC2219382

[20] Oaks MK Hallett KM Penwell RT Stauber EC Warren SJ Tector AJ A native soluble form of CTLA-4. Cell Immunol (2000) 201:144–53 10.1006/cimm.2000.1649 10831323

[21] Vijayakrishnan L Slavik JM Illes Z Greenwald RJ Rainbow D Greve B An autoimmune disease-associated CTLA-4 splice variant lacking the B7 binding domain signals negatively in T cells. Immunity (2004) 20:563–75. 10.1016/S1074-7613(04)00110-4 15142525

[22] Harper K Balzano C Rouvier E Mattei MG Luziani MF Golstein P. CTLA-4 and CD28 activated lymphocyte molecules are closely related in both mouse and human as to sequence, message expression, gene structure, and chromosomal location. J Immunol (1991) 147:1037–44. 1713603

[23] Lindsten T Lee KP Harris ES Petryniak B Craighead N Reynolds PJ Characterization of CTLA-4 structure and expression on human T cells. J Immunol (1993) 151:3489–99. 8397258

[24] Perkins D Wang Z Donovan C He H Mark D Guan G Regulation of CTLA-4 expression during T cell activation. J Immunol (1996) 156:4154–9. 8666782

[25] Linsley PS Brady W Urnes M Grosmaire LS Damle NK Ledbetter JA. CTLA-4 is a second receptor for the B cell activation antigen B7. J Exp Med (1991) 174:561–9. 10.1084/jem.174.3.561 1714933 PMC2118936

[26] Linsley PS Greene JL Brady W Bajorath J Ledbetter JA Peach R. Human B7-1 (CD80) and B7-2 (CD86) bind with similar avidities but distinct kinetics to CD28 and CTLA-4 receptors. Immunity (1994) 1:793–801. 10.1016/S1074-7613(94)80021-9 7534620

[27] Balzano C Buonavista N Rouvier E Golstein P. CTLA-4 and CD28: similar proteins, neighbouring genes. Int J Cancer Suppl (1992) 7:28–32. 1330947

[28] Schwartz JC Zhang X Fedorov AA Nathenson SG Almo SC. Structural basis for co-stimulation by the human CTLA-4/B7-2 complex. Nature (2001) 410:604–8. 10.1038/35069112 11279501

[29] Stamper CC Zhang Y Tobin JF Erbe DV Ikemizu S Davis SJ Crystal structure of the B7-1/CTLA-4 complex that inhibits human immune responses. Nature (2001) 410:608–11. 10.1038/35069118 11279502

[30] Chen Z Zhou F Huang S Jiang T Chen L Ge L Association of cytotoxic T lymphocyte associated antigen-4 gene (rs60872763) polymorphism with Crohn’s disease and high levels of serum sCTLA-4 in Crohn’s disease. J Gastroenterol Hepatol (2011) 26:924–30. 10.1111/j.1440-1746.2011.06662.x 21251066

[31] Liu MF Wang CR Chen PC Fung LL. Increased expression of soluble cytotoxic T-lymphocyte-associated antigen-4 molecule in patients with systemic lupus erythematosus. Scand J Immunol (2003) 57:568–72. 10.1046/j.1365-3083.2003.01232.x 12791095

[32] Oaks MK Hallett KM. Cutting edge: a soluble form of CTLA-4 in patients with autoimmune thyroid disease. J Immunol (2000) 164:5015–8. 10.4049/jimmunol.164.10.5015 10799854

[33] Tai X Van Laethem F Pobezinsky L Guinter T Sharrow SO Adams A Basis of CTLA-4 function in regulatory and conventional CD4(+) T cells. Blood (2012) 119:5155–63. 10.1182/blood-2011-11-388918 22403258 PMC3369608

[34] Takahashi T Kuniyasu Y Toda M Sakaguchi N Itoh M Iwata M Immunologic self-tolerance maintained by CD25+CD4+ naturally anergic and suppressive T cells: induction of autoimmune disease by breaking their anergic/suppressive state. Int Immunol (1998) 10:1969–80. 10.1093/intimm/10.12.1969 9885918

[35] Thornton AM Shevach EM. CD4+CD25+ immunoregulatory T cells suppress polyclonal T cell activation in vitro by inhibiting interleukin 2 production. J Exp Med (1998) 188:287–96. 10.1084/jem.188.2.287 9670041 PMC2212461

[36] Collison LW Workman CJ Kuo TT Boyd K Wang Y Vignali KM The inhibitory cytokine IL-35 contributes to regulatory T-cell function. Nature (2007) 450:566–9. 10.1038/nature06306 18033300

[37] Tadokoro CE Shakhar G Shen S Ding Y Lino AC Maraver A Regulatory T cells inhibit stable contacts between CD4+ T cells and dendritic cells in vivo. J Exp Med (2006) 203:505–11. 10.1084/jem.20050783 16533880 PMC2118249

[38] Tang Q Bluestone JA. The Foxp3+ regulatory T cell: a jack of all trades, master of regulation. Nat Immunol (2008) 9:239–44. 10.1038/ni1572 18285775 PMC3075612

[39] Lu Y Schneider H Rudd CE. Murine regulatory T cells differ from conventional T cells in resisting the CTLA-4 reversal of TCR stop-signal. Blood (2012) 120:4560–70. 10.1182/blood-2012-04-421420 23047820 PMC3557398

[40] Leach DR Krummel MF Allison JP. Enhancement of antitumor immunity by CTLA-4 blockade. Science (1996) 271:1734–6. 10.1126/science.271.5256.1734 8596936

[41] Attia P Phan GQ Maker AV Robinson MR Quezado MM Yang JC Autoimmunity correlates with tumor regression in patients with metastatic melanoma treated with anti-cytotoxic T-lymphocyte antigen-4. J Clin Oncol (2005) 23:6043–53. 10.1200/JCO.2005.06.205 16087944 PMC1473965

[42] Hodi FS Mihm MC Soiffer RJ Haluska FG Butler M Seiden MV Biologic activity of cytotoxic T lymphocyte-associated antigen 4 antibody blockade in previously vaccinated metastatic melanoma and ovarian carcinoma patients. Proc Natl Acad Sci U S A (2003) 100:4712–7. 10.1073/pnas.0830997100 12682289 PMC153621

[43] Phan GQ Yang JC Sherry RM Hwu P Topalian SL Schwartzentruber DJ Cancer regression and autoimmunity induced by cytotoxic T lymphocyte-associated antigen 4 blockade in patients with metastatic melanoma. Proc Natl Acad Sci U S A (2003) 100:8372–7. 10.1073/pnas.1533209100 12826605 PMC166236

[44] Ribas A Camacho LH Lopez-Berestein G Pavlov D Bulanhagui CA Millham R Antitumor activity in melanoma and anti-self responses in a phase I trial with the anti-cytotoxic T lymphocyte-associated antigen 4 monoclonal antibody CP-675,206. J Clin Oncol (2005) 23:8968–77. 10.1200/JCO.2005.01.109 16204013

[45] Grosso JF Jure-Kunkel MN. CTLA-4 blockade in tumor models: an overview of preclinical and translational research. Cancer Immun (2013) 13:5. 23390376 PMC3559193

[46] Weber JS Kahler KC Hauschild A. Management of immune-related adverse events and kinetics of response with ipilimumab. J Clin Oncol (2012) 30:2691–7. 10.1200/JCO.2012.41.6750 22614989

[47] Holmgaard RB Zamarin D Munn DH Wolchok JD Allison JP. Indoleamine 2,3-dioxygenase is a critical resistance mechanism in antitumor T cell immunotherapy targeting CTLA-4. J Exp Med (2013) 210:1389–402. 10.1084/jem.20130066 23752227 PMC3698523

[48] Robert L Tsoi J Wang X Emerson R Homet B Chodon T CTLA4 blockade broadens the peripheral T-cell receptor repertoire. Clin Cancer Res (2014) 20:2424–32. 10.1158/1078-0432.CCR-13-2648 24583799 PMC4008652

[49] Prieto PA Yang JC Sherry RM Hughes MS Kammula US White DE CTLA-4 blockade with ipilimumab: long-term follow-up of 177 patients with metastatic melanoma. Clin Cancer Res (2012) 18:2039–47. 10.1158/1078-0432.CCR-11-1823 22271879 PMC3319861

[50] Mamalis A Garcha M Jagdeo J. Targeting the PD-1 pathway: a promising future for the treatment of melanoma. Arch Dermatol Res (2014) 306:511–9. 10.1007/s00403-014-1457-7 24615548 PMC4110159

[51] Weber J. Immune checkpoint proteins: a new therapeutic paradigm for cancer – preclinical background: CTLA-4 and PD-1 blockade. Semin Oncol (2010) 37:430–9. 10.1053/j.seminoncol.2010.09.005 21074057

[52] Francisco LM Sage PT Sharpe AH. The PD-1 pathway in tolerance and autoimmunity. Immunol Rev (2010) 236:219–42. 10.1111/j.1600-065X.2010.00923.x 20636820 PMC2919275

[53] Ise W Kohyama M Nutsch KM Lee HM Suri A Unanue ER CTLA-4 suppresses the pathogenicity of self antigen-specific T cells by cell-intrinsic and cell-extrinsic mechanisms. Nat Immunol (2010) 11:129–35. 10.1038/ni.1835 20037585 PMC3235641

[54] Bachmann MF Kohler G Ecabert B Mak TW Kopf M. Cutting edge: lymphoproliferative disease in the absence of CTLA-4 is not T cell autonomous. J Immunol (1999) 163:1128–31. 10415006

[55] Masteller EL Chuang E Mullen AC Reiner SL Thompson CB. Structural analysis of CTLA-4 function in vivo. J Immunol (2000) 164:5319–27. 10.4049/jimmunol.164.10.5319 10799894

[56] Wang CJ Kenefeck R Wardzinski L Attridge K Manzotti C Schmidt EM Cutting edge: cell-extrinsic immune regulation by CTLA-4 expressed on conventional T cells. J Immunol (2012) 189:1118–22. 10.4049/jimmunol.1200972 22753931 PMC3442233

[57] Chuang E Fisher TS Morgan RW Robbins MD Duerr JM Vander Heiden MG The CD28 and CTLA-4 receptors associate with the serine/threonine phosphatase PP2A. Immunity (2000) 13:313–22. 10.1016/S1074-7613(00)00031-5 11021529

[58] Schneider H Smith X Liu H Bismuth G Rudd CE. CTLA-4 disrupts ZAP70 microcluster formation with reduced T cell/APC dwell times and calcium mobilization. Eur J Immunol (2008) 38:40–7. 10.1002/eji.200737423 18095376 PMC5580795

[59] Pentcheva-Hoang T Egen JG Wojnoonski K Allison JP. B7-1 and B7-2 selectively recruit CTLA-4 and CD28 to the immunological synapse. Immunity (2004) 21:401–13. 10.1016/j.immuni.2004.06.017 15357951

[60] Chikuma S Imboden JB Bluestone JA. Negative regulation of T cell receptor-lipid raft interaction by cytotoxic T lymphocyte-associated antigen 4. J Exp Med (2003) 197:129–35. 10.1084/jem.20021646 12515820 PMC2193802

[61] Darlington PJ Baroja ML Chau TA Siu E Ling V Carreno BM Surface cytotoxic T lymphocyte-associated antigen 4 partitions within lipid rafts and relocates to the immunological synapse under conditions of inhibition of T cell activation. J Exp Med (2002) 195:1337–47. 10.1084/jem.20011868 12021313 PMC2193751

[62] Martin M Schneider H Azouz A Rudd CE. Cytotoxic T lymphocyte antigen 4 potently inhibits cell surface raft expression in its regulation of T cell function. J Exp Med (2001) 194:1675–81. 10.1084/jem.194.11.1675 11733581 PMC2193535

[63] Rudd CE Martin M Schneider H. CTLA-4 negative signaling via lipid rafts: a new perspective. Sci STKE (2002) 2002:E18. 11972356 10.1126/stke.2002.128.pe18

[64] Rudd CE Schneider H. Unifying concepts in CD28, ICOS and CTLA4 co-receptor signalling. Nat Rev Immunol (2003) 3:544–56. 10.1038/nri1131 12876557

[65] Schneider H Prasad KVS Shoelson SE Rudd CE. CTLA-4 binding to the lipid kinase phosphatidylinositol 3-kinase in T cells. J Exp Med (1995) 181:351–5. 10.1084/jem.181.1.351 7807015 PMC2191832

[66] Cilio CM Daws MR Malashicheva A Sentman CL Holmberg D. Cytotoxic T lymphocyte antigen 4 is induced in the thymus upon in vivo activation and its blockade prevents anti-CD3-mediated depletion of thymocytes. J Exp Med (1998) 188:1239–46. 10.1084/jem.188.7.1239 9763603 PMC2212496

[67] Marengère LEM Waterhouse P Duncan GS Mittrücker H-W Feng G-S Mak TW. Regulation of T cell receptor signaling by tyrosine phosphatase Syp association with CTLA-4. Science (1996) 272:1170–3. 10.1126/science.272.5265.1170 8638161

[68] Chuang E Alegre M-L Duckett CS Noel PJ Vander Heiden MG Thompson CB. Interaction of CTLA-4 with the clathrin-associated protein AP50 results in ligand-independent endocytosis that limits cell surface expression. J Immunol (1997) 159:144–51. 9200449

[69] Schneider H Martin M Agarraberes FA Yin L Rapoport I Kirchhausen T Cytolytic T lymphocyte-associated antigen-4 and the TcRz/CD3 complex, but not CD28, interact with clathrin adaptor complexes AP-1 and AP-2. J Immunol (1999) 163:1868–79. 10438921

[70] Zhang Y Allison JP. Interaction of CTLA-4 with AP-50, a clathrin-coated pit adaptor protein. Proc Natl Acad Sci U S A (1997) 94:9273–8. 10.1073/pnas.94.17.9273 9256472 PMC23153

[71] Teft WA Chau TA Madrenas J. Structure-function analysis of the CTLA-4 interaction with PP2A. BMC Immunol (2009) 10:23. 10.1186/1471-2172-10-23 19405949 PMC2683795

[72] Parry RV Chemnitz JM Frauwirth KA Lanfranco AR Braunstein I Kobayashi SV CTLA-4 and PD-1 receptors inhibit T-cell activation by distinct mechanisms. Mol Cell Biol (2005) 25:9543–53. 10.1128/MCB.25.21.9543-9553.2005 16227604 PMC1265804

[73] Boasso A Herbeuval JP Hardy AW Winkler C Shearer GM. Regulation of indoleamine 2,3-dioxygenase and tryptophanyl-tRNA-synthetase by CTLA-4-Fc in human CD4+ T cells. Blood (2005) 105:1574–81. 10.1182/blood-2004-06-2089 15466932

[74] Fallarino F Grohmann U Hwang KW Orabona C Vacca C Bianchi R Modulation of tryptophan catabolism by regulatory T cells. Nat Immunol (2003) 4:1206–12. 10.1038/ni1003 14578884

[75] Munn DH Mellor AL. Indoleamine 2,3 dioxygenase and metabolic control of immune responses. Trends Immunol (2013) 34:137–43. 10.1016/j.it.2012.10.001 23103127 PMC3594632

[76] Schmidt SV Schultze JL. New insights into IDO biology in bacterial and viral infections. Front Immunol (2014) 5:384. 10.3389/fimmu.2014.00384 25157255 PMC4128074

[77] Mellor AL Munn D Chandler P Keskin D Johnson T Marshall B Tryptophan catabolism and T cell responses. Adv Exp Med Biol (2003) 527:27–35 10.1007/978-1-4615-0135-0_3 15206713

[78] Oida T Xu L Weiner HL Kitani A Strober W. TGF-beta-mediated suppression by CD4+CD25+ T cells is facilitated by CTLA-4 signaling. J Immunol (2006) 177:2331–9. 10.4049/jimmunol.177.4.2331 16887994

[79] Sullivan TJ Letterio JJ van Elsas A Mamura M van Amelsfort J Sharpe S Lack of a role for transforming growth factor-b in cytotoxic T lymphocyte antigen-4-mediated inhibition of T cell activation. Proc Natl Acad Sci USA (2001) 98:2587–92. 10.1073/pnas.051632398 11226283 PMC30182

[80] Letterio JJ Geiser AG Kulkarni AB Dang H Kong L Nakabayashi T Autoimmunity associated with TGF-beta1-deficiency in mice is dependent on MHC class II antigen expression. J Clin Invest (1996) 98:2109–19. 10.1172/JCI119017 8903331 PMC507656

[81] Ramsdell F Ziegler SF. FOXP3 and scurfy: how it all began. Nat Rev Immunol (2014) 14:343–9. 10.1038/nri3650 24722479

[82] Wing K Onishi Y Prieto-Martin P Yamaguchi T Miyara M Fehervari Z CTLA-4 control over Foxp3+ regulatory T cell function. Science (2008) 322:271–5. 10.1126/science.1160062 18845758

[83] Wing K Yamaguchi T Sakaguchi S. Cell-autonomous and -non-autonomous roles of CTLA-4 in immune regulation. Trends Immunol (2011) 32:428–33. 10.1016/j.it.2011.06.002 21723783

[84] Qureshi OS Zheng Y Nakamura K Attridge K Manzotti C Schmidt EM Trans-endocytosis of CD80 and CD86: a molecular basis for the cell-extrinsic function of CTLA-4. Science (2011) 332:600–3. 10.1126/science.1202947 21474713 PMC3198051

[85] Kong KF Fu G Zhang Y Yokosuka T Casas J Canonigo-Balancio AJ Protein kinase C-η controls CTLA-4-mediated regulatory T cell function. Nat Immunol (2014) 15:465–72. 10.1038/ni.2866 24705298 PMC4040250

[86] Yokosuka T Kobayashi W Takamatsu M Sakata-Sogawa K Zeng H Hashimoto-Tane A Spatiotemporal basis of CTLA-4 costimulatory molecule-mediated negative regulation of T cell activation. Immunity (2010) 33:326–39 10.1016/j.immuni.2010.09.006 20870175

[87] Rudd CE. The reverse stop-signal model for CTLA4 function. Nat Rev Immunol (2008) 8:153–60. 10.1038/nri2253 18219311

[88] Schneider H Downey J Smith A Zinselmeyer BH Rush C Brewer JM Reversal of the TCR stop signal by CTLA-4. Science (2006) 313:1972–5 10.1126/science.1131078 16931720

[89] Dillon TJ Carey KD Wetzel SA Parker DC Stork PJ. Regulation of the small GTPase Rap1 and extracellular signal-regulated kinases by the costimulatory molecule CTLA-4. Mol Cell Biol (2005) 25:4117–28. 10.1128/MCB.25.10.4117-4128.2005 15870282 PMC1087740

[90] Schneider H Valk E Dias S Wei B Rudd CE. CTLA-4 up-regulation of lymphocyte function-associated antigen 1 adhesion and clustering as an alternate basis for coreceptor function. Proc Natl Acad Sci U S A (2005) 102:12861–6. 10.1073/pnas.0505802102 16126897 PMC1192824

[91] Hara S Nakaseko C Yamasaki S Hattori M Bos JL Saito Y Involvement of Rap-1 activation and early termination of immune synapse in CTLA-4-mediated negative signal. Hematology (2009) 14:150–8. 10.1179/102453309X402241 19490760

[92] Knieke K Hoff H Maszyna F Kolar P Schrage A Hamann A CD152 (CTLA-4) determines CD4 T cell migration in vitro and in vivo. PLoS One (2009) 4:e5702. 10.1371/journal.pone.0005702 19479036 PMC2682661

[93] Mustelin T Immunology. Restless T cells sniff and go. Science (2006) 313:1902–3 10.1126/science.1133578 17008518

[94] Ruocco MG Pilones KA Kawashima N Cammer M Huang J Babb JS Suppressing T cell motility induced by anti-CTLA-4 monotherapy improves antitumor effects. J Clin Invest (2012) 122:3718–30. 10.1172/JCI61931 22945631 PMC3461908

[95] Lozanoska-Ochser B Klein NJ Huang GC Alvarez RA Peakman M. Expression of CD86 on human islet endothelial cells facilitates T cell adhesion and migration. J Immunol (2008) 181:6109–16. 10.4049/jimmunol.181.9.6109 18941200

[96] Linsley PS Bradshaw J Greene J Peach R Bennet KL Mittler RS. Intracellular trafficking of CTLA-4 and focal localization towards sites of TCR engagement. Immunity (1996) 4:535–43. 10.1016/S1074-7613(00)80480-X 8673700

[97] Mead KI Zheng Y Manzotti CN Perry LC Liu MK Burke F Exocytosis of CTLA-4 is dependent on phospholipase D and ADP ribosylation factor-1 and stimulated during activation of regulatory T cells. J Immunol (2005) 174:4803–11. 10.4049/jimmunol.174.8.4803 15814706

[98] Blumberg RS Ley S Sancho J Lonberg N Lacy E McDermott F Structure of the T-cell antigen receptor: evidence for two CD3 epsilon subunits in the T-cell receptor-CD3 complex. Proc Natl Acad Sci U S A (1990) 87:7220–4. 10.1073/pnas.87.18.7220 2144901 PMC54715

[99] Rutledge T Cosson P Manolios N Bonifacino JS Klausner RD. Transmembrane helical interactions: zeta chain dimerization and functional association with the T cell antigen receptor. EMBO J (1992) 11:3245–54. 1505516 10.1002/j.1460-2075.1992.tb05402.x PMC556858

[100] Kliche S Lindquist JA Schraven B. Transmembrane adapters: structure, biochemistry and biology. Semin Immunol (2004) 16:367–77. 10.1016/j.smim.2004.08.017 15541652

[101] Lindquist JA Simeoni L Schraven B. Transmembrane adapters: attractants for cytoplasmic effectors. Immunol Rev (2003) 191:165–82. 10.1034/j.1600-065X.2003.00007.x 12614359

[102] Bruyns E Marie-Cardine A Kirchgessner H Sagolla K Shevchenko A Mann M T cell receptor (TCR) interacting molecule (TRIM), a novel disulfide-linked dimer associated with the TCR-CD3-zeta complex, recruits intracellular signaling proteins to the plasma membrane. J Exp Med (1998) 188:561–75. 10.1084/jem.188.3.561 9687533 PMC2212462

[103] Zhu M Granillo O Wen R Yang K Dai X Wang D Negative regulation of lymphocyte activation by the adaptor protein LAX. J Immunol (2005) 174:5612–9. 10.4049/jimmunol.174.9.5612 15843560

[104] Kölsch U Arndt B Reinhold D Lindquist JA Juling N Kliche S Normal T-cell development and immune functions in TRIM-deficient mice. Mol Cell Biol (2006) 26:3639–48. 10.1128/MCB.26.9.3639-3648.2006 16612002 PMC1447406

[105] Kirchgessner H Dietrich J Scherer J Isomaki P Korinek V Hilgert I The transmembrane adaptor protein TRIM regulates T cell receptor (TCR) expression and TCR-mediated signaling via an association with the TCR zeta chain. J Exp Med (2001) 193:1269–84. 10.1084/jem.193.11.1269 11390434 PMC2193385

[106] Shapiro MJ Nguyen CT Aghajanian H Zhang W Shapiro VS. Negative regulation of TCR signaling by linker for activation of X cells via phosphotyrosine-dependent and -independent mechanisms. J Immunol (2008) 181:7055–61. 10.4049/jimmunol.181.10.7055 18981125 PMC2630470

[107] Zhu M Janssen E Leung K Zhang W. Molecular cloning of a novel gene encoding a membrane-associated adaptor protein (LAX) in lymphocyte signaling. J Biol Chem (2002) 277:46151–8. 10.1074/jbc.M208946200 12359715

[108] Valk E Leung R Kang H Kaneko K Rudd CE Schneider H. T cell receptor-interacting molecule acts as a chaperone to modulate surface expression of the CTLA-4 coreceptor. Immunity (2006) 25:807–21. 10.1016/j.immuni.2006.08.024 17070077

[109] Valk E Rudd CE Schneider H CTLA-4 trafficking and surface expression. Trends Immunol (2008) 29:272–9 10.1016/j.it.2008.02.011 18468488 PMC4186961

[110] Banton MC Inder KL Valk E Rudd CE Schneider H. Rab8 binding to immune cell-specific adaptor LAX facilitates formation of trans-Golgi network-proximal CTLA-4 vesicles for surface expression. Mol Cell Biol (2014) 34:1486–99. 10.1128/MCB.01331-13 24515439 PMC3993577

[111] Dietrich J Kastrup J Lauritsen JP Menne C von Bulow F Geisler C. TCRzeta is transported to and retained in the Golgi apparatus independently of other TCR chains: implications for TCR assembly. Eur J Immunol (1999) 29:1719–28. 10.1002/(SICI)1521-4141(199905)29:05<1719::AID-IMMU1719>3.3.CO;2-D 10359127

[112] Pfeffer S Aivazian D. Targeting Rab GTPases to distinct membrane compartments. Nat Rev Mol Cell Biol (2004) 5:886–96. 10.1038/nrm1500 15520808

[113] Stenmark H. Rab GTPases as coordinators of vesicle traffic. Nat Rev Mol Cell Biol (2009) 10:513–25. 10.1038/nrm2728 19603039

[114] Zerial M McBride H Rab proteins as membrane organizers. Nat Rev Mol Cell Biol (2001) 2:107–17 10.1038/35052055 11252952

[115] Peränen J Auvinen P Virta H Wepf R Simons K. Rab8 promotes polarized membrane transport through reorganization of actin and microtubules in fibroblasts. J Cell Biol (1996) 135:153–67. 10.1083/jcb.135.1.153 8858170 PMC2121014

[116] Peränen J Furuhjelm J Expression, purification, and properties of Rab8 function in actin cortical skeleton organization and polarized transport. Methods Enzymol (2001) 329:188–96 10.1016/S0076-6879(01)29079-X 11210535

[117] Hattula K Furuhjelm J Tikkanen J Tanhuanpaa K Laakkonen P Peränen J. Characterization of the Rab8-specific membrane traffic route linked to protrusion formation. J Cell Sci (2006) 119:4866–77. 10.1242/jcs.03275 17105768

[118] Nagabhushana A Chalasani ML Jain N Radha V Rangaraj N Balasubramanian D Regulation of endocytic trafficking of transferrin receptor by optineurin and its impairment by a glaucoma-associated mutant. BMC Cell Biol (2010) 11:4. 10.1186/1471-2121-11-4 20085643 PMC2826298

[119] Dong C Yang L Zhang X Gu H Lam ML Claycomb WC Rab8 interacts with distinct motifs in alpha2B- and beta2-adrenergic receptors and differentially modulates their transport. J Biol Chem (2010) 285:20369–80. 10.1074/jbc.M109.081521 20424170 PMC2888448

[120] Beraud-Dufour S Balch W A journey through the exocytic pathway. J Cell Sci (2002) 115:1779–80. 11956309 10.1242/jcs.115.9.1779

[121] D’Souza-Schorey C Chavrier P ARF proteins: roles in membrane traffic and beyond. Nat Rev Mol Cell Biol (2006) 7:347–58 10.1038/nrm1910 16633337

[122] Freyberg Z Siddhanta A Shields D. “Slip, sliding away”: phospholipase D and the Golgi apparatus. Trends Cell Biol (2003) 13:540–6. 10.1016/j.tcb.2003.08.004 14507482

[123] Carrasco S Merida I. Diacylglycerol, when simplicity becomes complex. Trends Biochem Sci (2007) 32:27–36. 10.1016/j.tibs.2006.11.004 17157506

[124] Shemesh T Luini A Malhotra V Burger KN Kozlov MM. Prefission constriction of Golgi tubular carriers driven by local lipid metabolism: a theoretical model. Biophys J (2003) 85:3813–27. 10.1016/S0006-3495(03)74796-1 14645071 PMC1303683

[125] Takahashi T Tagami T Yamazaki S Uede T Shimizu J Sakaguchi N Immunologic self-tolerance maintained by CD25(+)CD4(+) regulatory T cells constitutively expressing cytotoxic T lymphocyte-associated antigen 4. J Exp Med (2000) 192:303–10. 10.1084/jem.192.2.303 10899917 PMC2193248

[126] Bradshaw JD Lu P Leytze G Rodgers J Schieven GL Bennett KL Interaction of the cytoplasmic tail of CTLA-4 (CD152) with a clathrin-associated protein is negatively regulated by tyrosine phosphorylation. Biochemistry (1997) 36:15975–82. 10.1021/bi971762i 9398332

[127] Shiratori T Miyatake S Ohno H Nakaseko C Isono K Bonifacino JS Tyrosine phosphorylation controls internalization of CTLA-4 by regulating its interaction with clathrin-associated adaptor complex AP-2. Immunity (1997) 6:583–9. 10.1016/S1074-7613(00)80346-5 9175836

[128] Miyatake S Nakaseko C Umemori H Yamamoto T Saito T. Src family tyrosine kinases associate with and phosphorylate CTLA-4 (CD 152). Biochem Biophys Res Commun (1998) 249:444–8. 10.1006/bbrc.1998.9191 9712716

[129] Schneider H Schwartzberg PL Rudd CE. Resting lymphocyte kinase (Rlk/Txx) phosphorylates the YVKM motif and regulates PI 3-kinase binding to T-cell antigen CTLA-4. Biochem Biophys Res Commun (1998) 252:14–9. 10.1006/bbrc.1998.9559 9813138

[130] Guntermann C Alexander DR. CTLA-4 suppresses proximal TCR signaling in resting human CD4(+) T cells by inhibiting ZAP-70 Tyr(319) phosphorylation: a potential role for tyrosine phosphatases. J Immunol (2002) 168:4420–9. 10.4049/jimmunol.168.9.4420 11970985

[131] Kozik P Francis RW Seaman MN Robinson MS. A screen for endocytic motifs. Traffic (2010) 11:843–55. 10.1111/j.1600-0854.2010.01056.x 20214754 PMC2882754

[132] Iida T Ohno H Nakaseko C Sakuma M Takeda-Ezaki M Arase H Regulation of cell surface expression of CTLA-4 by secretion of CTLA-4-containing lysosomes upon activation of CD4+ T cells. J Immunol (2000) 165:5062–8. 10.4049/jimmunol.165.9.5062 11046036

[133] Egen JG Allison JP. Cytotoxic T lymphocyte antigen-4 accumulation in the immunological synapse is regulated by TCR signal strength. Immunity (2002) 16:23–35. 10.1016/S1074-7613(01)00259-X 11825563

[134] Jago CB Yates J Camara NO Lechler RI Lombardi G. Differential expression of CTLA-4 among T cell subsets. Clin Exp Immunol (2004) 136:463–71. 10.1111/j.1365-2249.2004.02478.x 15147348 PMC1809051

